# Tactile sensing biohybrid soft E-skin based on bioimpedance using aloe vera pulp tissues

**DOI:** 10.1038/s41598-021-82549-x

**Published:** 2021-02-04

**Authors:** Mostafa A. Mousa, MennaAllah Soliman, Mahmood A. Saleh, Ahmed G. Radwan

**Affiliations:** 1 Nanoelectronics Integrated Systems Center (NISC), Nile University, Sheikh Zayed City, 12588 Egypt; 2 Mechanical Engineering Program, School of Engineering and Applied Sciences, Nile University, Sheikh Zayed City, 12588 Egypt; 3 Department of Engineering Mathematics and Physics, Cairo University, Giza, 12613 Egypt; 4 School of Engineering and Applied Sciences, Nile University, Sheikh Zayed City, 12588 Egypt

**Keywords:** Electrical and electronic engineering, Mechanical engineering

## Abstract

Soft and flexible E-skin advances are a subset of soft robotics field where the soft morphology of human skin is mimicked. The number of prototypes that conformed the use of biological tissues within the structure of soft robots—to develop “Biohybrid Soft Robots”—has increased in the last decade. However, no research was conducted to realize Biohybrid E-skin. In this paper, a novel biohybrid E-skin that provides tactile sensing is developed. The biohybrid E-skin highly mimics the human skin softness and morphology and can sense forces as low as 0.01 newton . The tactile sensing feature is augmented through the use of Aloe Vera pulp, embedded in underlying channel, where the change in its bioimpedance is related to the amount of force exerted on the E-skin surface. The biohybrid E-skin employs high biomimicry as the sensorial output is an oscillating signal similar to signals sent from the human sensing neurons to the brain. After investigating different channel geometries, types of filling tissues, and usage of two silicone materials, their frequency-force behaviour is modelled mathematically. Finally, a functional multichannel prototype “ImpEdded Skin” is developed. This prototype could efficiently detect the position of a tactile touch. This work employs the development of discrete sensing system that exhibits morphological computation that consequently enhances performance.

## Introduction

Since the evolution of robots and its emergence in most of today’s industrial, medical, agriculture, and exploratory applications, many kinds of research have been deployed to create a shared working environment between humans and robots^1^. However, these efforts have always faced difficulties owing to the hard nature of the robots’ rigid links. Soft bio-inspired robotics is a new domain in robotics that offer new opportunities to expand the scope of robot applications and create a safe, shared environment between robots and us. Hard robots are missing features as collaborating, simplicity, lower cost, lightweight, and thermodynamic efficiency that soft robots possess^2^.

Soft robots are defined as systems equipped with autonomous behavior that exploited materials with moduli in the range of soft biological ones^3^. Since the rise of the soft robotics domain in robotics science, many pieces of research have been conducted to realize robot actuators^4^, sensors^5^, and even controllers^6,7^ in soft matter.

Soft biohybrid robots are a subset of the soft robotics domain which uses biological tissues within its structure. The use of biological tissues within a soft robot has first been presented in 2007^8^. Since then, studies have been deployed to investigate the potential of this new approach in building soft robots with respect to actuation, sensing^9^, and control^10^. Bio hybrid soft robots combine the best features among the biological systems and artificial ones. It provides entirely new aspects as self-healing of robots, sense soft touch, or to adapt to new environments. The integration between these two systems enables the realization of untethered functions depending on cells’ chemical energy^10^. Embedding living materials in soft robots have more advantages being lightweight as well as it requires little energy for actuation. Soft biohybrid robots are driven by the integration of different disciplines in one interdisciplinary paradigm^11^. In our case, material science, bioimpedance, pneumatics, and analog electronics are integrated in one interdisciplinary application.

Soft robots have enhanced the biomimicry in robotics; multiple works have been presented to mimic muscular hydrostats of living organisms like octopus^12^, worms^13^, fish^14^, and elephant trunks as their soft structures able to bend, twist and extend^15^. The knowledge of the morphology and function of soft structures encourages new designs of soft robotics mimicry biological structure. Scientists inspire new flexible materials from the natural to improve soft robotics. Thanks to the flexibility of the materials used to fabricate soft robots, soft robots have surpassed hard robots in such applications related to biomimicry.

E-skin is one form of bio-mimicry in soft robotics where the morphology of human skin is being mimicked^16^. Skin for living organisms protects its inner organs and provide them with sensing capabilities through its different receptors. The receptors situated under the skin’s outermost layer (epithelium), are located at different depth. Therefore, developing E-skin prototypes involved using a variety of sensors to enable real-time sensing-processing. This paved the way for E-skin to be used in the field of robotics and biomedical applications. On one hand it could enhance robots’ sensing^17,18^ abilities for better Human Robot Interaction (HRI). On the other hand, its features was explored in the field of smart prosthetics^19,20^. However, these reported prototypes are not the best approach to mimic the skin’s soft morphology as they use rigid components—sensors. Thus, our proposed prototype the mechanoreceptors are mimicked by a soft gel-like biological tissue that is embedded at a certain depth under the E-skin surface.

**Figure 1:**
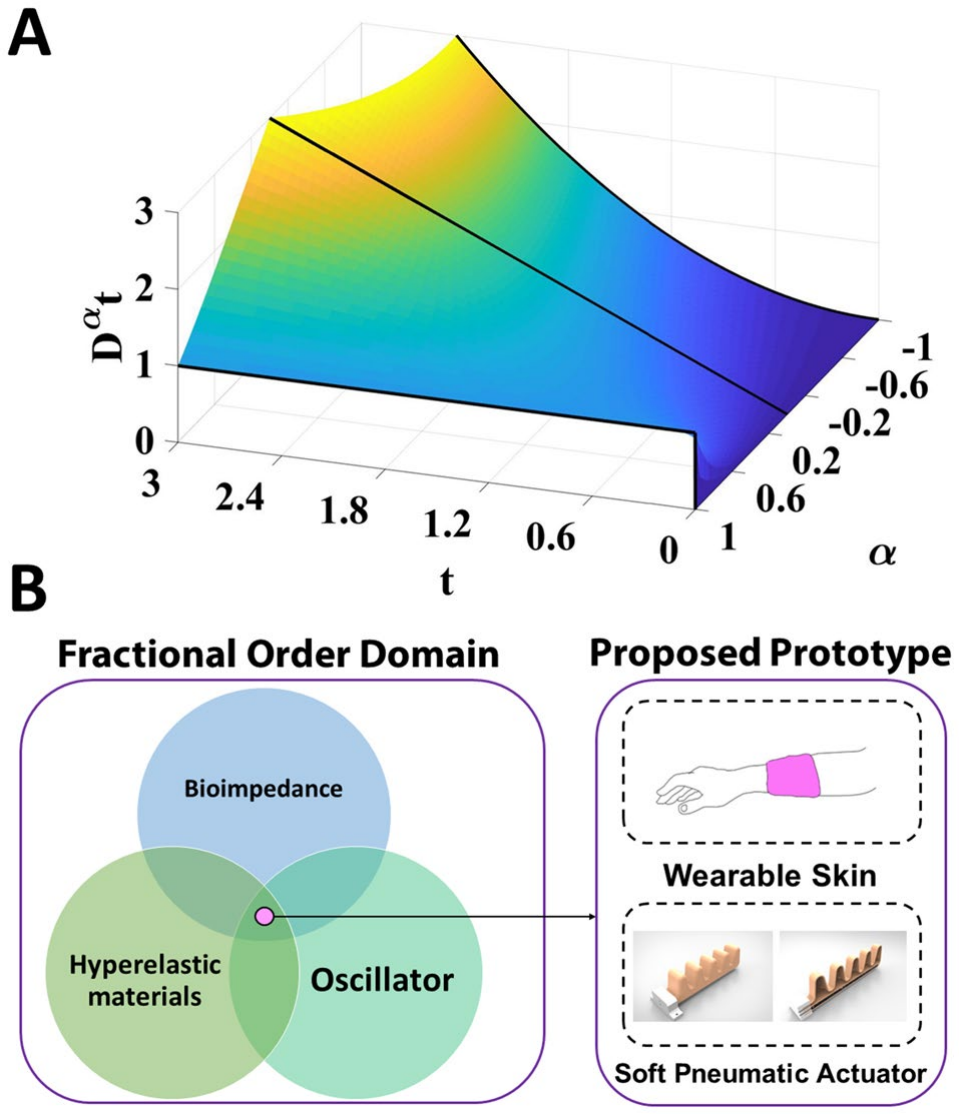
Fractional order domain and the proposed prototype. (**A**) The integral of step function throughout different fractional orders. (**B**) The proposed work is based on three main pillars; Biological impedance of tissues, hyper elastic materials and oscillators. These pillars are common for their realization in the context of the fractional order domain, and upon them our proposed prototype is based. Adding channels beneath a soft E-skin or embedded inside a soft pneumatic actuator which are filled by biological soft tissues can augment tactile sensing capability based on changes of the tissues bioimpedance.

### Bioimpedance, morphological computation, and biohybrid E-skin realization

Sensing through biological materials is briefly discussed and demonstrated. The cornerstone of this work findings is Bioimpedance. Bioimpedance is the resistance of tissue to an applied AC signal. Conduction in biological tissues depend on ionic presence and its intensity depends on ionic content density and mobility^21^. This how conductivity, and so on impedance, are related to the cellular organelles and composition. Correlating the physical state of tissues to electric parameters enables the non-destructive inspection of different stress or quality traits.

Bioimpedance has been used heavily in medical applications^22^, and there are recent advances in other fields like agriculture^23^. Bioimpedance is researched for monitoring the growth of plants like strawberries^24^, yeast, and mandarin. It also has been investigated to differentiate the aggressiveness of cancer cells based on growth dynamics in conjunction with Bio-impedimetric analysis^25^.

Bioimpedance, hyper-elastic materials, and oscillators are the three main pillars upon which the proposed prototype is developed. These pillars are interpreted in the fractional-order domain. Fractional order calculus is a branch of mathematics that was discovered by the two scientists Leibnitz and L’Hopital. Throughout the last years, fractional-order calculus has proved to be of great impact in many applications^26^. Fractional order calculus has recently been deployed in many applications and showed huge throughput in terms of modelling^27^. There are multiple definitions for fractional order differentiation. Caputo is one of the commonly used definitions and it is expressed as^28^:

1 $$_{\alpha }^{C} D_{t}^{\alpha } f(t) = \frac{1}{{\Gamma (m - \alpha )}}\int\limits_{a}^{t} {(t - \tau )^{{m - \alpha - 1}} f^{{(m)}} (\tau )d\tau } ,$$

whereas $m-1 \le \alpha \le m$ and $m \in \mathbb {N}$ and its laplace representation is:

2 $${\mathcal{L}} \left\{ {_{\alpha }^{C} D_{t}^{\alpha } f(t)} \right\} = s^{\alpha } F(s) - \sum\nolimits_{{k = 0}}^{{m - 1}} {s^{{\alpha - k - 1}} D^{k} f(0)} .$$

The visualization of the fractional-order derivative of a ramp function with respect to different $\alpha$ values is shown in Fig. 1A.

Interpretation and analysis of tissues’ impedance behavior have been facilitated since the use of fractional order calculus^29^. Multiple models have been introduced to model the bioimpedance of tissues. These models varied between integer and fractional order models. Fractional order models had proved to be more accurate and enabled better understanding and precise modeling of biological tissues. Also, hyperelastic materials have been modeled using fractional order differential equations^30^. Thanks to finite element fractional capacitor approximation methods, the realization of fractional order-based oscillators is possible. The classical marginal stability oscillator design equations are generalized to the fractional-order domain -when using fractional-order elements-^27^.

In this work, bioimpedance is applied in a new area and discover its potential in such a rising domain. The potential of bioimpedance to enhance biohybrid soft robots prototypes and realize biohybrid E-skin was previously presented^31^. The use of bioimpedance to capture the change of force applied to a biological tissue embedded in a channel inside E-skin or a soft robot is proposed, as in Fig.1B. Force applied to E-skin fabricated from a hyper-elastic material like silicone rubber leads to the deformation of the channel carrying the biological tissue, which changes its overall impedance. Impedance changes can be recorded by multiple direct and indirect methods. Direct methods are based on using dedicated devices to measure impedance while, indirect methods conform the using of optimization techniques and parameters extraction methods^32,33^. The change in biological tissue impedance, which is a component in a Single Resistance Controlled Oscillator (SRCO) circuit, changes the output frequency of the oscillator.

“Offloading computations from brain to body” concept have been conceived lately in robots and soft robots design and control^34^. This concept ,which is commonly known by morphological computation, goal is to reduce the burden on the robotics main controller by replicating some of its functions through the robots own body. With respect to this work’s contribution in the tactile sensing domain, morphological computation is defined as any method that utilises the mechanical and geometrical characteristics of the robot body to facilitate perception^35^. In other words, the body itself in form of the flexible structure is contributing to the tactile sensing feature. Thus, in this work, a discrete sensing system that employs morphological computation—where the E-skin deformation is directly translated to an oscillating signal that consequently enhances performance- is proposed.

The outcome of this paper was achieved through two main experiments. The first experiment had been designed to study the relation between the applied force to a soft structure and the change in bioimpedance of underlying soft tissue. Soft structures (E-skin samples) fabricated from commercial silicone rubber were designed with different internal cavities that creates different channels. Multiple channel’s cross-section geometries had been investigated in this experiment; round shape, and oval-like shape with respect to two different orientations. Moreover, the soft structure was fabricated using two commercial silicone rubber products of two different shore hardness. These distinct structures were fabricated through molding in a 3D printed mold. The biological tissue under test in this experiment was Aloe Vera. The use of Aloe Vera pulp solely and a mixture of Aloe Vera pulp with gelatin had been investigated. Thus, in this experiment, the variance of materials used in the fabrication of soft structure, and the effect of gelatin as a medium when combined with Aloe Vera pulp is investigated. Based on these results, a biohybrid E-skin prototype is fabricated. Finally, The multichannel biohybrid E-skin ability to record the position of touch is validated experimentally.

## Methods

### Bioimpedance and applied force characterization experiment

**Figure 2:**
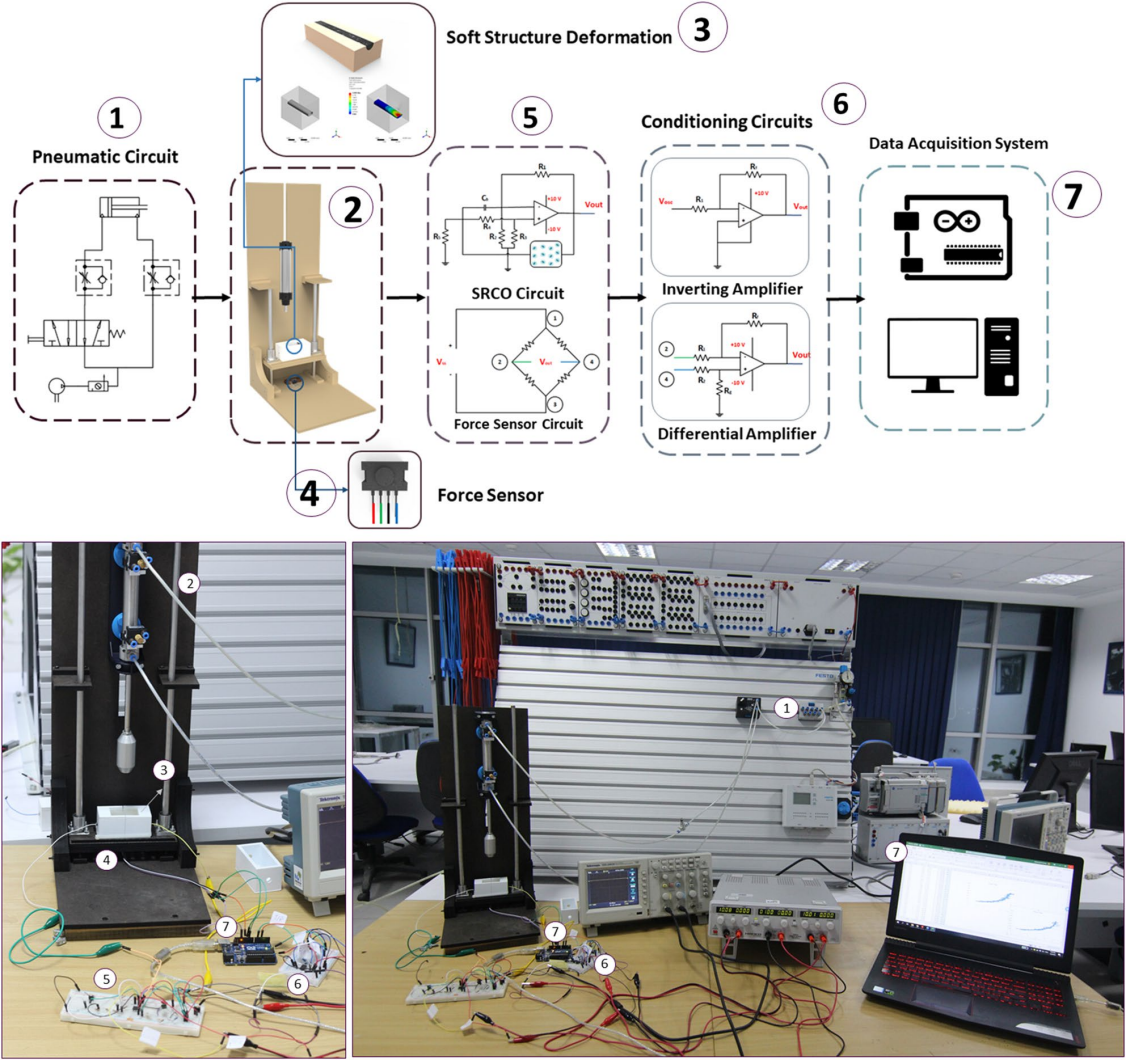
Block Diagram of experimental characterization. The source of force in this experiment is a double acting cylinder controlled by the represented pneumatic circuit (1). The cylinder is fixed to a mechanical setup (2) where the silicone structure (3) is placed. The two variables; force acquired by the force sensor (4) and change in bioimpedance acquired in the form of change of output frequency of SRCO circuit (5), undergo two signal conditioning circuits (6) before being acquired by the data acquisition circuit (7).

The experiment was designed to apply force gradually at a slow and constant rate on a soft E-skin structure that includes the sensing biological material. This was achieved through a double-acting cylinder which stroke is $8$ mm in diameter. It was placed in a pneumatic circuit controlled through a 5/2 FESTO pneumatic valve and two FESTO one-way controlled flow valves. The soft structure was then placed in a 3D printed plastic case inside a slider mechanism (see Supplementary Figs. S1–S2) placed over a Honeywell high precision force sensor, which has a resolution of 0.0098 N. The block diagram in Fig. 2 shows the complete setup of the characterization experiment.

The pneumatic circuit was supplied by compressed air, and the stroke of the double-acting cylinder was stretched out until it touches the soft silicone structure. Then it started exerting force on its surface, increasing the readings of the force sensor, and the change in bioimpedance was recorded through the SRCO circuit. Both values undergo signal conditioning circuits before being recorded by the data acquisition system.

The Aloe Vera was bought as a plant pot from the local market. The Aloe Vera leaf was cut from its roots, and then the pulp is separated from its rind. After separation, the pulp was transformed to the gel-like from by being cut using a standard blender. In case using Aloe Vera only as of the soft tissue, the preparation process ends by this step. While when using Aloe Vera-Gelatin mixture, powder gelatin bought from the local market was dissolved into warm water and then mixed with Aloe Vera at a ratio of 1:1 (see Supplementary Fig. S6–S7).

**Figure 3:**
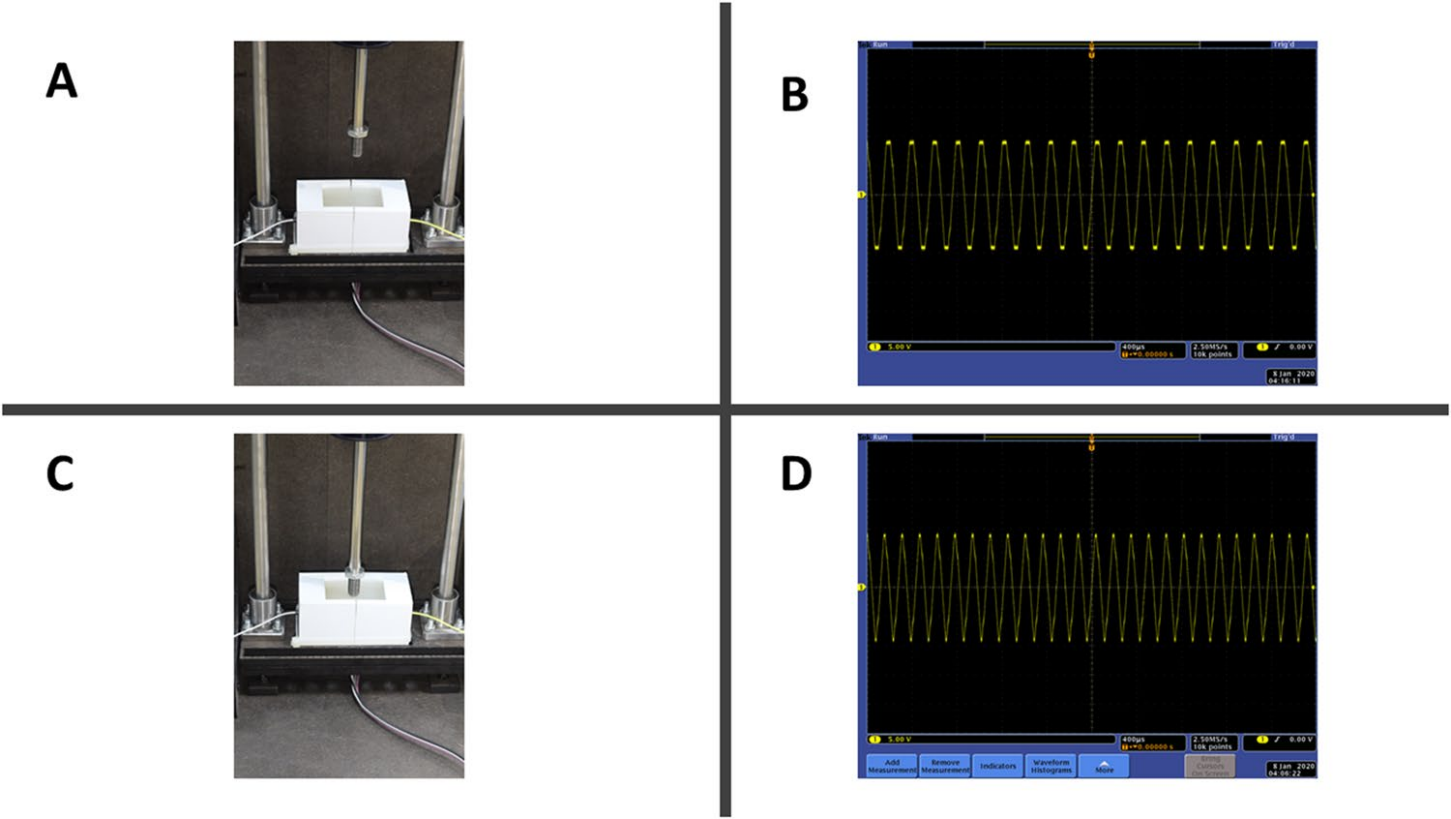
View of the experimental setup and the experiment results. In (**A**) the stroke is stretching yet did not reach the surface of the soft silicone rubber structure. The oscillation in (**B**) shows to be around 4 kHz before the contact moment. (**C**) shows after loading force on the soft structure leading to a rise in the frequency recorded as clear in (**D**).

**Figure 4:**
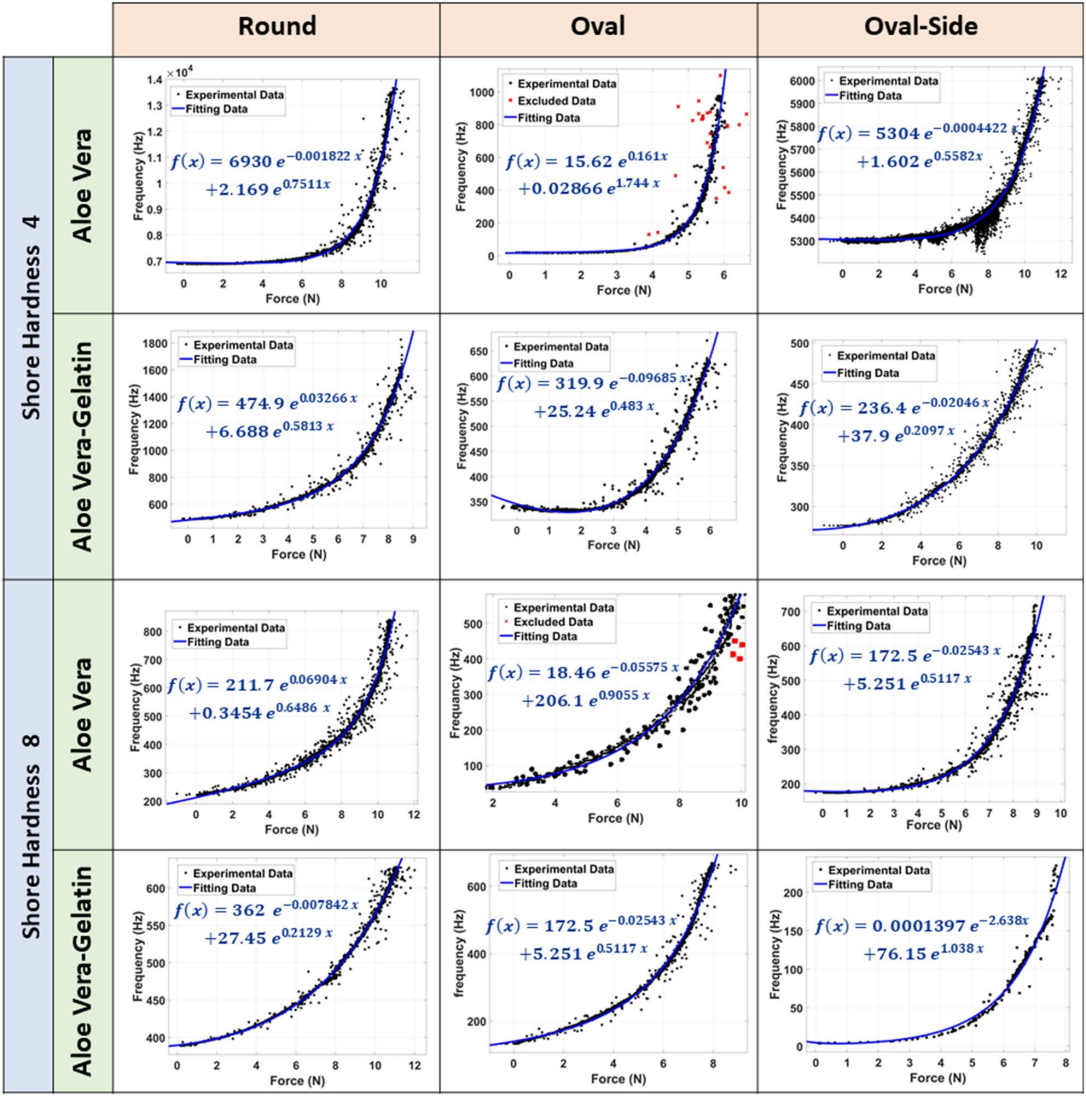
Relation between an applied force and bioimpedance expressed in terms of frequency. The results pf the two tissues used in this experiment—Aloe Vera and Aloe Vera-Gelatin mixture—are shown. The behavior of these tissues is investigated in different channel geometries and different shore hardness silicone material. The fitting results are added to each graph and the fitting equation that is an exponential function is represented on each result.

The silicone rubber structures were filled with the soft gel-like biological tissue and then were encapsulated inside a plastic holder (see Supplementary Fig. S3) that covers all its faces except for the top face. The plastic holder has two more functions; preventing leakage of the gel-like tissue and connecting the tissue with two electrodes. The plastic holder keeps the two electrodes positions constant throughout the experiment, which was crucial for the regeneration of the experiment. The plastic holder was then placed into a mechanical slider setup that allows applying vertical force to the top face of the silicone structure. The mechanical setup was designed with a slot for a very high-resolution force sensor for recording the applied force values. The force applied to the soft structure ranged from no-load (0 Newtons) till the failure of the oscillator circuit—that occurs when the channel was completely closed—which was around 10–12 Newtons.

The second parameter measured in this experiment is the overall impedance of the soft biological tissue impeded in the channel within the silicone structure. Bioimpedance is usually measured by impedance analyzers that come at different specifications. Multiple research was conducted to realize low cost and portable impedance analyzers. Nevertheless, in this work, the change of impedance was measured in-directly through an oscillator circuit. The SRCO output oscillation frequency is proportional to the overall impedance of the inspected biological tissue. To sum up, applying force to the silicone structure leads to the deformation of the internal channel geometry, which consequently changes the impedance of the gel-like tissue, finally changing the oscillation frequency, the variation in the SRCO output frequency is shown in Fig. 3.

The result of this experiment is a relation between the applied force to the silicone structure and the oscillating frequency of an SRCO circuit. The relation was derived for both silicone structures fabricated at shore hardness 4 and 8, and three different channel geometries for each structure. The results showed consistency, and the resultant curves behaviour showed to match exponential function curve. Thus, the experimental data points were fitted to two-terms exponential function (3) where *x* is the force in Newton and *f*(*x*) is the frequency in Hertz. The scale coefficients of the equation are (*a*, *c*), while (*b*, *d*) are curve coefficients. The force measured from the force sensor was considered as input of the function that calculates the frequency of bio-impedance. The equations that describe the relationship between force and frequency are shown in Fig. 4.

3 $$\begin{aligned} f(x) = ae^{bx} + c e^{dx}. \end{aligned}$$

### Soft structure, molds and connectors fabrication

**Figure 5:**
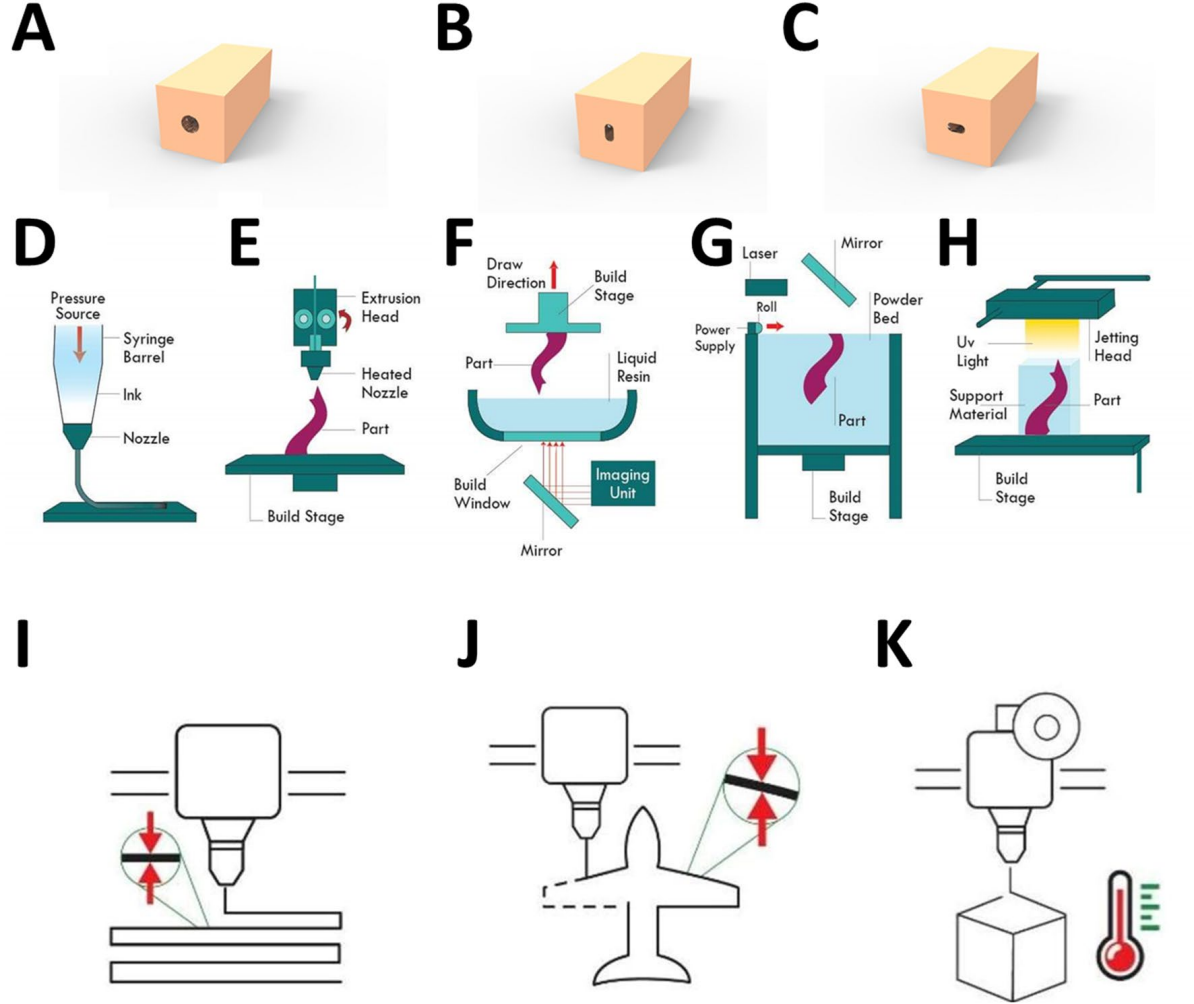
Channels Geometry and additive manufacturing technologies. In (**A**), (**B**) and (**C**) the designed geometries of the channels are shown, taking into consideration that the force is applied from upward direction. While (**D**–**H**) shows the principle of the most famous 3D printers; direct ink writing, fused deposition modeling, stereolithography, selective laser sintering, and inkjet printing. Finally, an illustration visualizing the parameters optimized is shown for (**I**) layer height, (**J**) line width and (**K**) printing temperature.

The soft structure used in the experiment was fabricated using Zhermack commercial silicon rubber (ZA 4 LT ROSSO, and ZA 8 LT). These two products have shore hardness 4, and 8 respectively. A suitable amount of base material and hardener were poured in a container and mixed using a plastic stick for 10 min and were left to solidify for 75 min. The mixture then was poured inside a 3D-printed mold and was left to solidify. The ratio of the base material to the hardener is 1:1. This process is shown in Supplementary Fig. S5. The cross section of the inner channel dimension is as follows; for the cylindrical channel the circular cross-section is of 8 mm in diameter. While for the other channel it is 8 mm in length, 4 mm in width and the for corners are completely filleted to form an oval-like shape. The illustration of the soft structures and the channel geometry is shown in Fig. 5A–C.

Additive manufacturing technologies have facilitated rapid prototyping and in-lab manufacturing recently. 3D printing is one form of digital additive manufacturing that enables fast and efficient fabrication of complex models. In this work, 3D printing has been utilized to print all the molds and connectors used in the experiments.

3D printing is realized based on various and diverse fabrication techniques. The simplest and most used technique is Fused Deposition Modeling (FDM) shown in Fig.5E. A filament polymer is extruded to a hot tiny nozzle which deposits the melted polymer on a platform to solidify instantaneously. FDM technology is popular for its low cost and various materials used like Acrylonitrile Butadiene Styrene (ABS), Polylactic Acid (PLA), nylon and polycarbonate. Another similar printing technology to FDM is the Direct Ink Writing (DIW) method shown in Fig.5D. It follows the same technique where the additive material flows from a nozzle in liquid form and instantly solidifies after being selectively extruded. The difference between them is that DIW is based on s a liquid ink of a polymeric precursor and is forced out from the nozzle using a plunger rather than the extruded filament in case of FDM.

The trade-off of the cheap and simple technology like FDM is less accuracy and precision compared to other sophisticated 3d printing techniques like Stereo Lithography (SLA), Selective Laser Sintering (SLS), and direct inkjet printing (see Fig. 5F–H). SLA, and SLS are laser based techniques that reach an accuracy of $0.01$ mm layer height compared to $0.1$ mm in the case of FDM printers. Unlike FDM which only accepts filament-shaped materials, SLA, and SLS uses laser to cure and solidify liquid resin and powder polymer respectively^1^. While inkjet printing process is based on three main steps. The first one is direct deposition of ink droplets on a substrate. Afterwards, these drops interact with their neighbouring droplets. Finally, vitrification, evaporation and/or polymerization of the ink takes place leading to its solidification. However, in our application the resolution of an FDM printer was enough. Thus, Ultimaker 3D printer was used to print all the mold and connectors using PLA filament after slicing the design on Cura Software as shown in Supplementary Fig. S4.

Parameters optimization is crucial for a successful printing process. The commonly modified parameters are layer height, line width, and printing temperature as shown in Fig. 5I–K respectively. Layer height is the thickness of the deposited material in the Z-axis, while line width is the thickness in the 2D plan. Appropriate selection of printing temperature is crucial to avoid lack of adherence of layers in case of low printing temperature, and messy prints in case of high ones.

### Single resistance controlled oscillator (SRCO) circuit

**Figure 6:**
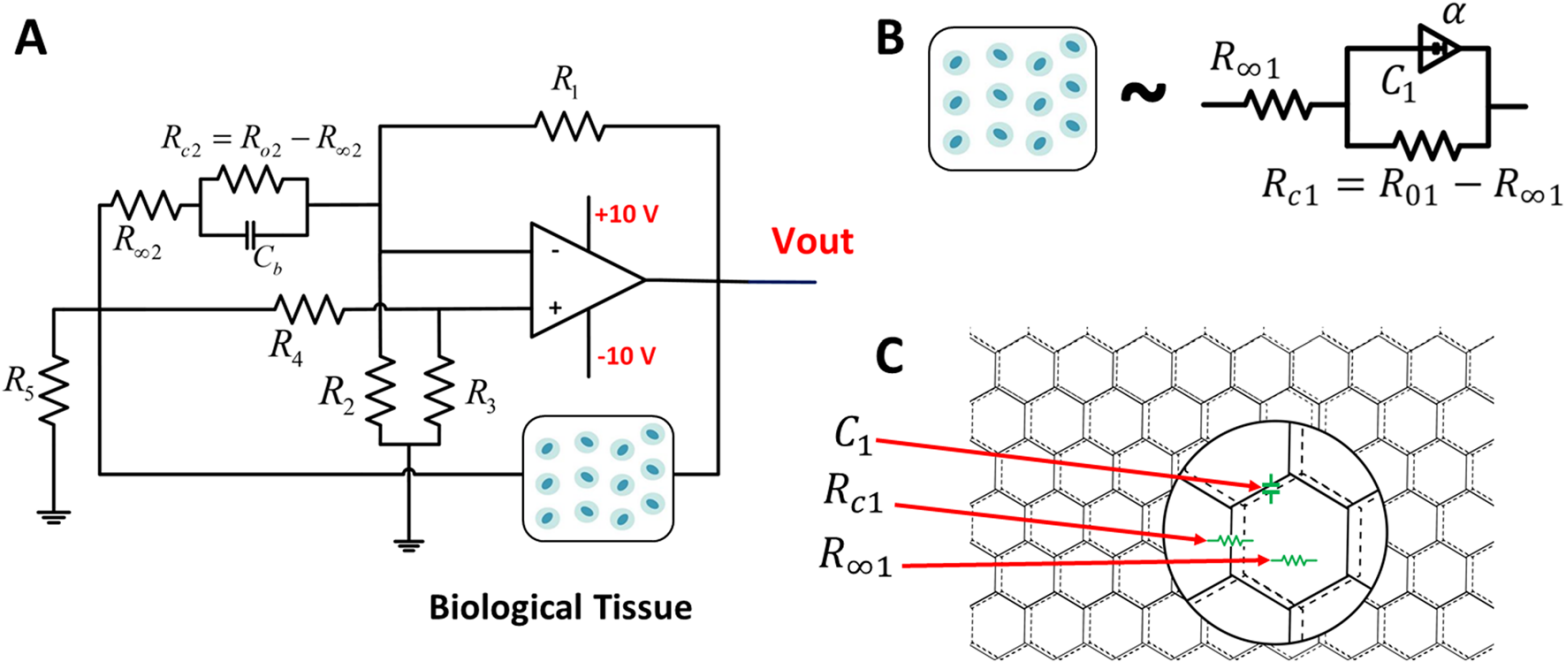
The modified SRCO circuit. (**A**) A parallel RC branch is replaced by the biological tissue. Many Models have been proposed to model the behavior of impedance introduced by various biological tissues. In (**B**) Single Dispersion Cole model which is equivalent to the biological tissue, and in (**C**) each parameter of the Cole model is related to the cell wall, and liquid gel of the actual Aloe Vera pulp tissue.

The oscillator circuit employed in this paper shown in Fig. 6 is different from conventional SRCO circuit. A parallel RC branch is replaced by the embedded biological tissue. This approach has been inspected in previously but in terms of optimization application^36^. The Cole model parameters each correspond to a physical structure or and organelle in the pulp tissue. The Aloe Vera pulp mainly consists of three main components; cell wall, liquid gel, and other micro-particles^37^. The Cole model parameters $R_{\infty 1}, R_{c1}$, and $C_1$ models the resistance of the liquid gel, resistance of the cell wall, and the capacitance between cell walls, respectively as shown in Fig. 6C.

The equation of the mentioned oscillator circuit derived through linear analysis is:

4 $$\begin{aligned}&21C_1^2R_{c1}(900.49\times 10^3R_{\infty 1}-12.14\times 10^7)s^{\alpha +1}+ C_1R_{c1}(44.7R_{\infty 1}-2.4)s^\alpha \nonumber \\&\quad + 21C_1(2.03R_{\infty 1}+2.03R_{c1}+0.2)s+ 4.47\times 10^{-6}R_{\infty 1}R_{c1}+44.7R_{\infty 1} +100\times 10^{-9}R_{c1}-2.4=0, \end{aligned}$$

and by substituting $s^\alpha =\omega ^\alpha [\cos (\frac{\alpha \pi }{2})+j\sin (\frac{\alpha \pi }{2})]$ in equation (4) the following two equations are derived:

5 $$\begin{aligned}Real &= 21C_1^2R_{c1}(900.49\times 10^3R_{\infty 1}-12.14\times 10^7)\omega ^{\alpha +1}\cos \left( \frac{(\alpha +1)\pi }{2}\right) + C_1R_{c1}(44.7R_{\infty 1}-2.4)\omega ^\alpha \cos \left( \frac{\alpha \pi }{2}\right) \nonumber \\&\quad + 4.47\times 10^{-6}R_{\infty 1}R_{c1}+44.7R_{\infty 1} +100\times 10^{-9}R_{c1}-2.4 = 0, \end{aligned}$$

6 $$\begin{aligned}Imag. &= 21C_1^2R_{c1}(900.49\times 10^3R_{\infty 1}-12.14\times 10^7)\omega ^{\alpha +1}\sin \left( \frac{(\alpha +1)\pi }{2}\right) + C_1R_{c1}(44.7R_{\infty 1}-2.4)\omega ^\alpha \sin \left( \frac{\alpha \pi }{2}\right) \nonumber \\&\quad + 21C_1(2.03R_{\infty 1}+2.03R_{c1}+0.2)\omega = 0. \end{aligned}$$

To sum up, the Cole model parameters of the Aloe Vera tissue used $C_1, \alpha , R_{c1}$, and $R_{\infty 1}$ directly changes the oscillation frequency $\omega$. When the channel filled with tissue experiences deformation, the impedance of the tissue changes and consequently the Cole model parameters change leading to change of the oscillator frequency.

The oscillator circuit parameters are; $R_4=1\;{\text {k}}\Omega , R_3=90\;{\text {k}}\Omega , R_1=30\;{\text {k}}\Omega , R_{o2}=1\;{\text {k}}\Omega$, and $R_{c2}=21\;{\text {k}}\Omega$. While $R_2$ and $R_5$ are tuned to control the oscillation frequency. Thus, $R_2$ and $R_5$ are placed in circuit as potentiometers. However, the process of frequency tuning can be implemented automatically using a microcontroller and digital potentiometers.

### Data acquisition

The oscillator and the force sensor output undergo two different conditional circuits. The oscillator output varies between + 10 and − 10 V. It then passes through the inverting amplifier designed at gain = 0.5 using a single supply. Thus, it could be readable by the data acquisition element. While the force sensor output is a differential voltage of two Wheatstone bridge terminals.

A differential amplifier is designed to deliver the voltage difference of the two pins to the data acquisition element, which is the Arduino Uno development board. The development board is chosen for its simplicity of use and its suitability for the current application. The Arduino Uno Board comes with 10-bit ADC, which maps its analog input to 5 volts by default. In order to increase its resolution,1 volt is supplied to its analog reference pin (AREF), so it maps the analog input to 1 volt instead of 5 volts. The ADC was used to measure the force values to the nearest hundredth digit. While the oscillator frequency is measured using FreqMeasure library, which was validated throughout all the trials using a Tektronix oscilloscope. The frequency measurements were recorded 10 times before being averaged to eliminate the false readings. The measurements are printed serially to the workstation monitor and then are imported to Matlab for plotting.

## Results

**Figure 7:**
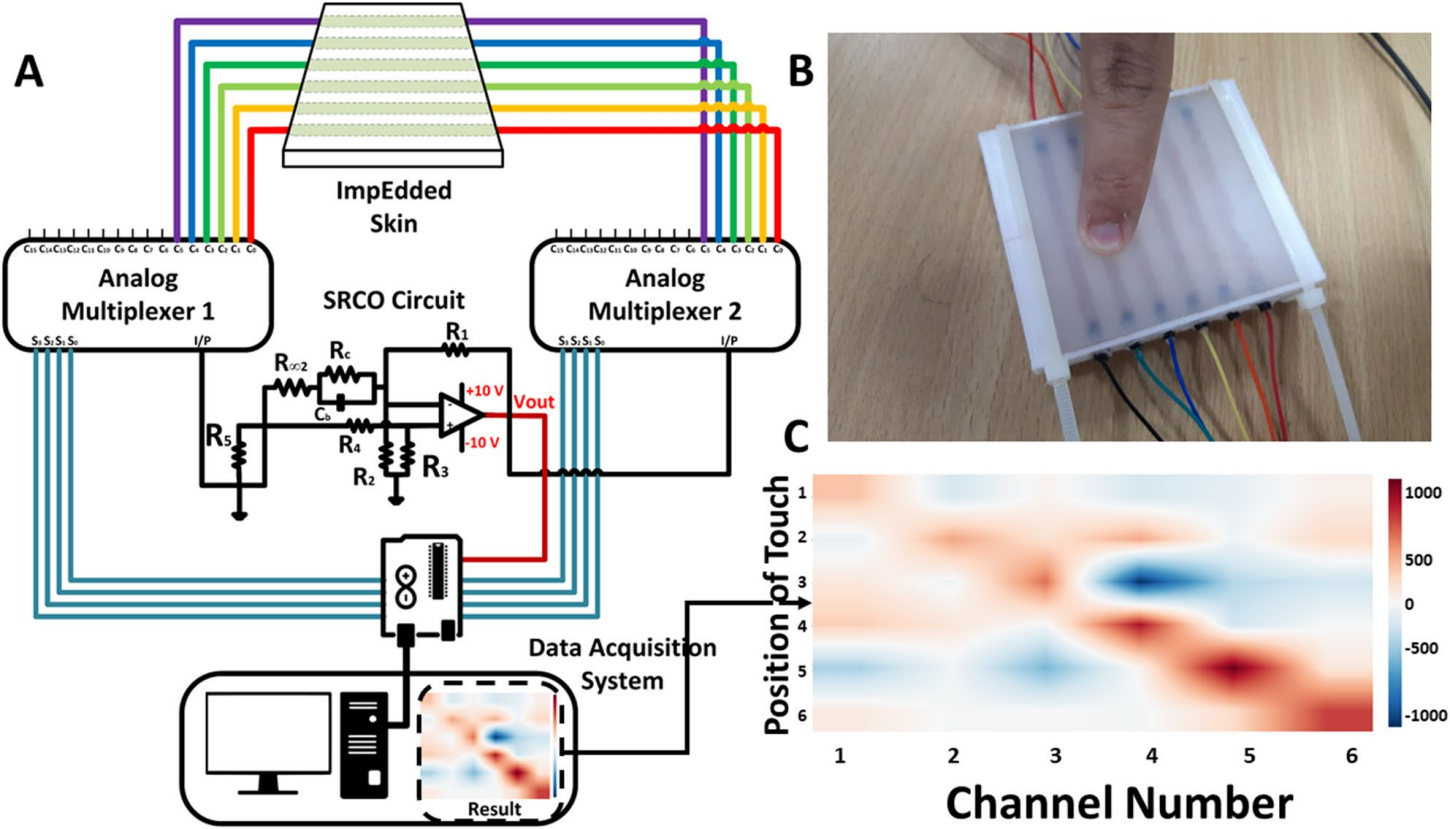
The ImpEdded skin experiment. (**A**) A special circuit is designed to acquire the frequency output from the SRCO circuit for each channel. Using two analog multiplexers and a microcontroller development board (Arduino UNO), switching between the six channels was possible. Then the frequency output for all the channels at each pressing iteration is readable. The data is acquired on a pc and the variation of frequencies before and after pressing is computed and represented on a heat map. (**B**) ImpEdded skin has six channels filled with Aloe Vera-gelatin mixture under pressing. (**C**) Each of the horizontal row represents an iteration of pressing on each channel, starting by the first channel at the first row and ending by the sixth channel at the bottom row, while each column represents the channels from left to right. The measurements on each tile represents the difference in frequency before and after pressing iteration.

The bioimpedance and applied force characterization experiment was conducted to explore three main variables. The first one was the tissue used to fill inside the channels, whether it is better to use Aloe Vera pulp or a mixture of it and gelatin. It was clear that a mixture of gelatin and Aloe Vera is more sensitive to the forces exerted on the surface of the soft silicone structure. This can be deducted from the rise of oscillation frequencies of the SRCO, which reflects a rise in the bioimpedance of the mixture at low force values. This was not the case where Aloe Vera pulp tissues only were used as the rise in oscillations begins after larger values of force exerted on the soft structure.

The second variable investigated is the channels’ cross-section geometry. The round cross-section achieves consistent measurements rather than the oval-like cross-section at its two orientations. This was obvious from the curve coefficients, which were in the same range for the two materials being used in fabrication. The last variable under investigation is the material used to fabricate the soft silicone structures. The silicone rubber of higher shore hardness could operate at higher force values. Thus, the silicone rubber of higher shore hardness was used to fabricate the proposed biohybrid E-skin prototype. It was designed to have channels of a round cross-section and is filled by a mixture of Aloe Vera pulp and gelatin.

**Table 1:** Summary of the Biohybrid soft robots, and the E-skin research mentioned in this work.

E-Skin				
References	Synthetic element	Biological element	Pressure range	Description
^38^	PU Elastomer	–	0–100 KPa	Tactile sensing, detects direction of force
^18^	CB silicone composite	–	0.5–38.2 MPa	High durable, tactile sensing skin
^19^	Stretchable dielectric, and stretchable ionic conductor	–	0–40 KPa	Skin works as pressure and strain sensor
^39^	Metal nanocparticles	–	NA	Apexcardiogram (ACG) sensor
^40^	Polyimine substrate, and polymer solution	–	NA	E-skin that has multiple sensing capabilities , can be recycled and rehealed
^20^	PDMS	–	0–1 KPa	E-skin for tactile sensing at extremely high sensitivity
This work	Silicone rubber	Aloe Vera Pulp	0–0.25 KPa	Highly sensitive biohybrid E-skin

### ImpEdded skin

After relating the applied force to the different samples and the impedance of the underlying soft tissue, a prototype was built to investigate the validity of the proposed methodology in sensing force with respect to different positions. A new multichannel biohybrid E-skin structure—ImpEdded Skin—was fabricated by molding. It was fabricated using commercial silicone rubber of shore hardness 8. The silicone rubber with higher shore hardness was selected owing to its enhanced behavior compared to the lower shore hardness one. It showed to exploit more linear behavior and better sensitivity at lower force measurements. The Aloe Vera–Gelatin mixture was chosen to be the soft tissue embedded inside the ImpEdded Skin. A plastic holder was designed on CAD tool, and 3D printed using FDM printer. It prevented leakage and connected the electrodes to the six channels. The ImpEdded Skin was designed to be thinner than the soft structure in the first experiment with smaller round channels of 5 mm diameter. The molds and connectors of the prototype is shown in Supplementary Fig. S8.

The E-skin structure after being filled and assembled, was then connected to the oscillator circuit through two analog multiplexers. And the frequencies were then recorded at the instances before and after touching. The differences between the frequencies were calculated and plotted as a heat map to show the frequency change in all channels at the instance of the touch of each channel, as in Fig. 7.

## Discussion

The results show a direct relationship between the applied force on channels inside a soft structure and the bioimpedance expressed in terms of oscillations. It also demonstrates how a prototype is built and the potential of the proposed methodology in realizing soft tactile sensing for E-skin through biohybrid paradigm which is not reported in literature yet. This paves the road towards the use of biological tissues in sensing applications for soft robotics like it has been used earlier in actuation^31^. The first experiment, which discusses the relation between the applied force and oscillation frequency, shows that the gelatin as a medium for Aloe Vera enhanced its behavior as the relationship became more linear in the different channels compared to using Aloe Vera solely. Moreover, the force-impedance relation is investigated at two different silicone rubber materials. It is clear how the channel geometry of the harder—higher shore hardness—material is deformed at lower force values. This can be observed at the slope of the curve at low force values. From a modeling point of view, this relation is expressed as an exponential function through fitting the acquired experimental data. The biological tissue impedance changes at $0.01$ N applied force which is considered satisfactory detection limit and with excellent resolution as shown in Fig. 4.

The ImpEdded Skin experiment shows the validity of the proposed solution to detect the position of tactile touch. The proposed prototype shows a good level of bio mimicry to the morphology of human skin. This is owing to the deformation of E-skin is the source of impedance variation. This physical change is transformed to change of oscillation frequency which is very close to actual signals sent from the human skin to the brain. This work is the first realization of biohybrid E-skin prototype. Comparison with other E-skin prototypes is shown in Table 1 where other novel prototypes are listed. The table shows that this work is the only one to conform the use of biological tissue within its structure. The table also shows it has a lower relative pressure range. Despite the fact that this range is sufficient for tactile sensing applications, this range is limited owing to the channel width and the material used. Higher shore hardness materials can exhibit higher pressure range as it can withstand higher forces without closing the underlying channel.

However, the use of biological tissues has its challenges as well. Biological tissues are unstable as the cells are in continuous movement, and after its extraction, they are in continuous aging till decomposition. This requires a continuous self-calibration technique, so the SRCO circuit continues to function throughout the tissue lifetime without acquiring false readings. This issue is one of the future work that would be addressed. A system including the proposed prototype integrated with a micro-controller and digital potentiometers can realize the automatic calibration feature. Another important aspect to be enhanced is the biohybrid system lifetime. The proposed prototype could function for 4 days till the complete decay of the Aloe Vera tissue. Using this tissue is not the most optimal choice, however it was used to proof the validity of the biohybrid sensing paradigm. Other alternatives are set to be explored in the future work like cell culturing and 3D printing of biological tissues (bio-printing). These techniques can be utilized to design particular tissues for sensing likewise for actuation^41,42^. Advances of biohybrid robotics can have a great impact in the field of healthcare, in terms of drug delivery and wearable devices.

The proposed sensing paradigm can be recognized as morphological sensing approach. By meshing the sensing methodology with the robot body, morphology is realized and perception is facilitated. The morphological perception in this system is primarily based on tactile sensing domain.

## Supplementary information

Supplementary Information.

Supplementary Legend.

Supplementary Video.
