# Supplementary material for: Tactile sensing biohybrid soft E-skin based on bioimpedance using aloe vera pulp tissues
Source: Sci Rep. 2021 Feb 4;11:3054. doi: 10.1038/s41598-021-82549-x (PMC7862233; doi:10.1038/s41598-021-82549-x)
Supplement: Supplementary file 1 — Supplementary Information. [file 41598_2021_82549_MOESM1_ESM.pdf]

SUPPLEMENTARY MATERIALS

Tactile Sensing Biohybrid Soft E-skin based on Bioimpedance using Aloe Vera Pulp Tissues

Authors

Mostafa A. Mousa[1,*], MennaAllah H. Soliman[2] , Mahmood A. Saleh[2] , and Ahmed G. Radwan[3,4]

1 Nanoelectronics Integrated Systems Center (NISC), Nile University, Sheikh Zayed City 12588, Egypt

2 Mechanical Engineering Program, School of Engineering and Applied Sciences, Nile University, Sheikh Zayed City 7 12588, Egypt

3 Department of Engineering Mathematics and Physics, Cairo University, Giza 12613, Egypt

4 School of Engineering and Applied Sciences, Nile University, Sheikh Zayed City 12588, Egypt

*Corresponding Author: mabdelrahman@nu.edu.eg

S1 Mechanical Setup

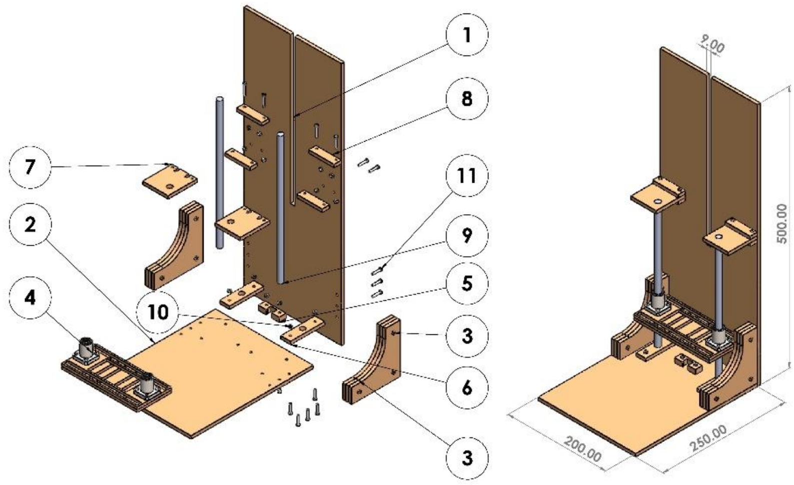

ITEM NO.	ITEM	QTY.
1	Vertical Plate	1
2	Horizontal Plate	1
3	Corner Set	2
4	Elevator set	1
5	Force Sensor Mount	1
6	Down End Support	2
7	Up end Support	2
8	Nut Cover	4
9	Linear Guide Rod 30 mm	2
10	M3 Hex Nut	14
11	M3 Screw 16 mm	22
12	M3 Screw 20 mm	4

Fig. S1. Illustration of mechanical system setup. The slider mechanical setup and its full part list.

A

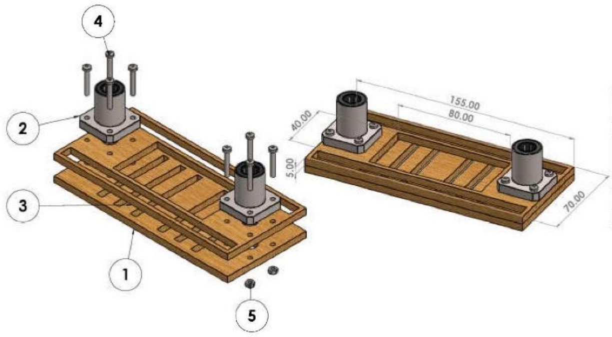

ITEM NO.	ITEM	QTY.
1	Elevator Base	1
2	Flange Linear Bearing 30 mm	2
3	Elevator Cavity	1
4	M3 Screw 20 mm	8
5	M3 Hex Nut	8

B

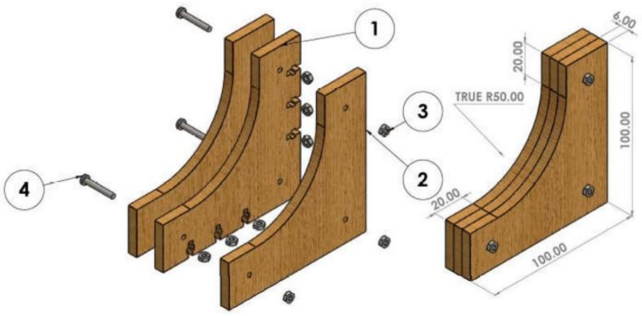

ITEM NO.	ITEM	QTY.
1	Nut Plate	1
2	Corner Plate	2
3	M3 Hex Nut	9
4	M3 Screw 20 mm	3

Fig. S2 Detailed assembly for corner and elevator set. (**A**) shows the elevator set assembly and the cavity were the sample under test is placed as well as (**B**) shows the proper assembly of the corner set using screws and nuts.

The mechanical setup shown in fig. S1 is designed using SolidWorks^{TM} CAD program and has been fabricated using high grade MDF wood bought from local markets. The assembly of the full setup is achieved using digital connection through screw and nuts. The setup is designed in order to attach a FESTO^{TM} double acting cylinder, to apply a force in slow and consistent manner. A very high-grade linear bearing is used to illuminate minor frictions. The elevator set shown in fig. S2 is designed with a cavity dedicated to including the soft structure enclosed in plastic holder concentric under the cylinder stroke.

S2 Plastic Holder

The plastic holder in fig. S3 is designed to be fabricated through additive manufacturing using a Fused Deposition Modeling (DFM) 3D printer. The channel width and shape were taken into consideration, and an inlet to the channel was designed to contain the contact wire with no leakage or exposing the tissue

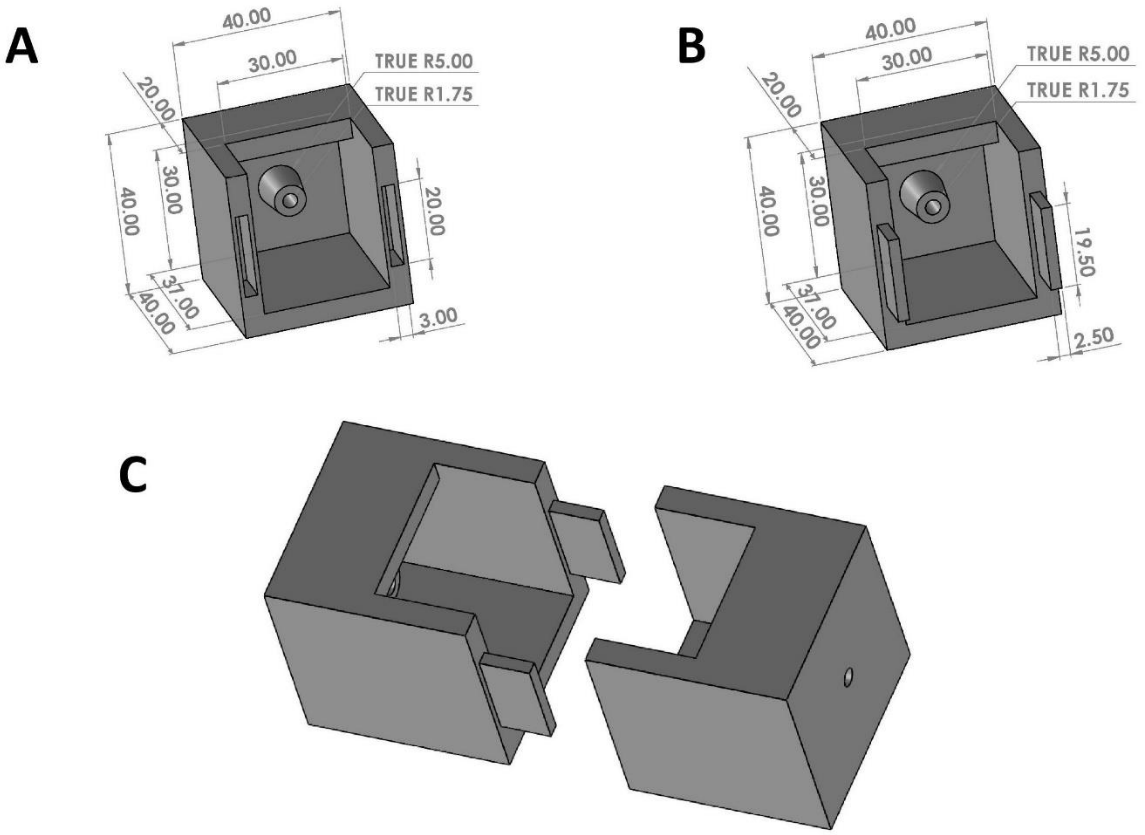

Fig. S3 Plastic Holder Design. The plastic holder design is split into two meshing parts; (**A**) female and (**B**) male. Their assembly is shown in (**C**).

S3 Preparation of Molds

Different molds to cast the soft structure with different channels cross section shown in fig. S4 and the ImpEdded Skin have been designed using SolidWorksTM CAD program. Then, the designs are converted to STL files to be 3D printed. The molds – similar to the plastic holders – are 3D printed using Ultimaker 2+, a Fused Deposition Modeling (FDM) 3D printer. The filament used is Flow™ Poly Lactic Acid (PLA) filament which is popular for its ease of use and good mechanical properties.

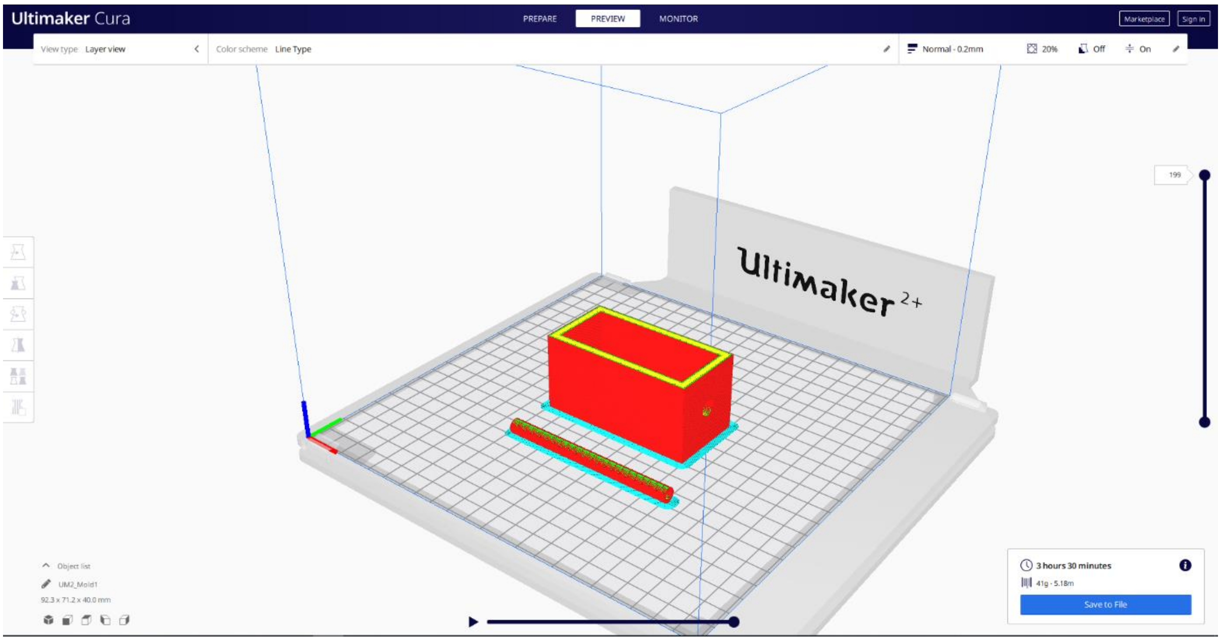

Fig. S4. Cura Software. Snapshot of the layered design of the designed mold from Cura Software.

The printing parameters are set to best quality standards: the layer height is 0.1 mm which provides the sense of continuity and smoothness. The shell of the printed part is of 1 mm in thickness, this is reasonable for this kind of applications. The printing temperature is determined by the manufacturer; in our case it is 200o C. other parameters are set to the default values; printing speed is 60 mm/sec, travel speed is 120 mm/sec, and support is enabled at low density. The software used to generate the G-Code out of the STL file is CURA™ as clear in fig. S4 which is the official software for Ultimakers 3D printers.

S4 Preparation of Silicon Rubber Based Soft Structure.

Figure S5 shows the mixture of silicon and its hardener. The silicon used is fabricated using ZhermackTM commercial silicon rubber (ZA 4 LT ROSSO, and ZA 8 LT). ZA 4 LT ROSSO is for shore hardness 4 and ZA 8 LT is shore hardness 8. Material is prepared by mix 1:1 for base material and hardening material. After steering the mixture is poured inside mold.

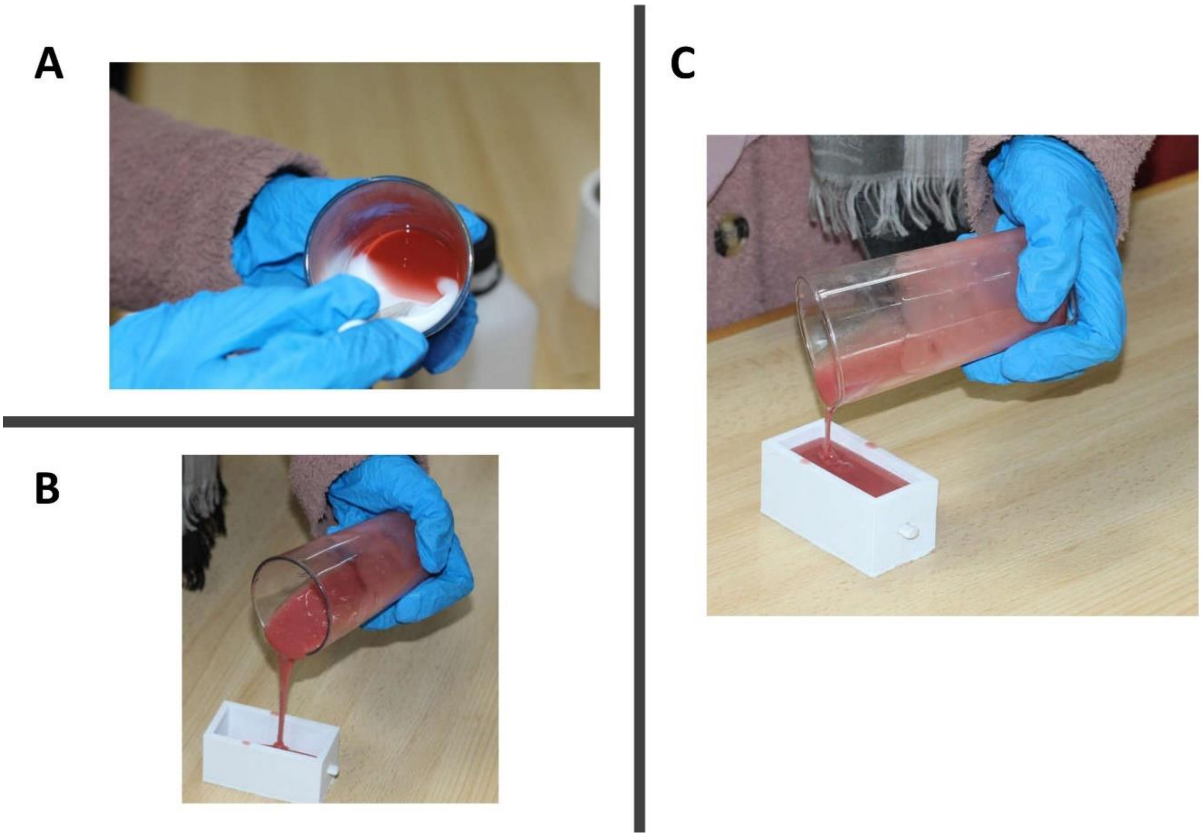

Fig. S5. Preparation of silicon rubber. (**A**) Mixture of base material and hardener before mixing. (**B**) Material after mixing. (**C**) pour the material in the mold.

S5 Preparation of Aloe Vera, and Aloe Vera-Gelatin Mixture.

The Aloe vera gel is extracted from leaves by separate pulp from its rind then blend pulp as shown in fig. S6. The powder gelatin is dissolved in warm water added to gel pulp to form Aloe Vera-Gelatin mixture in fig. S7.

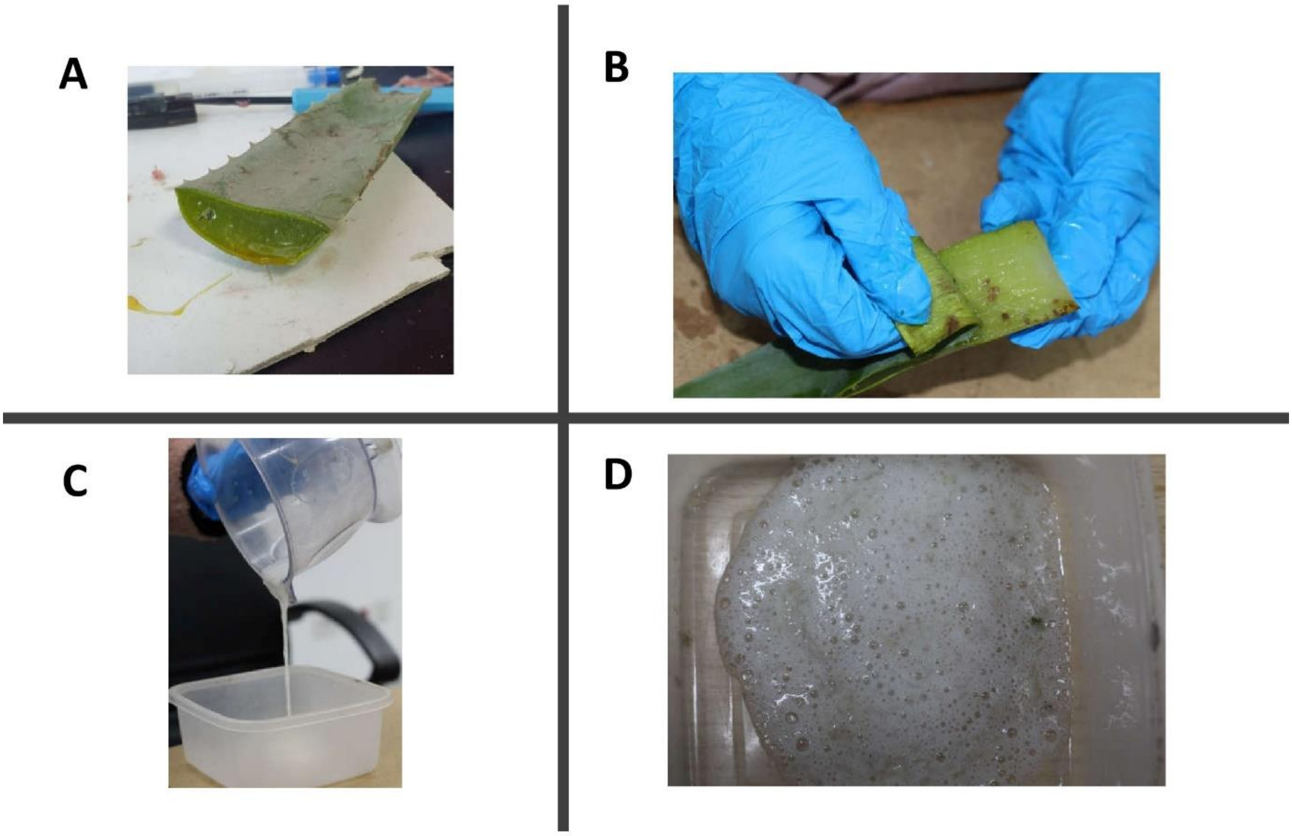

Fig. S6. Preparation of Aloe Vera. (**A**) Aloe Vera leaf cross section. (**B**) Extraction of Aloe Vera-Gel. (**C**) Grist of Aloe Vera Gel. (**D**) Aloe Vera mixture that fill cross section channel

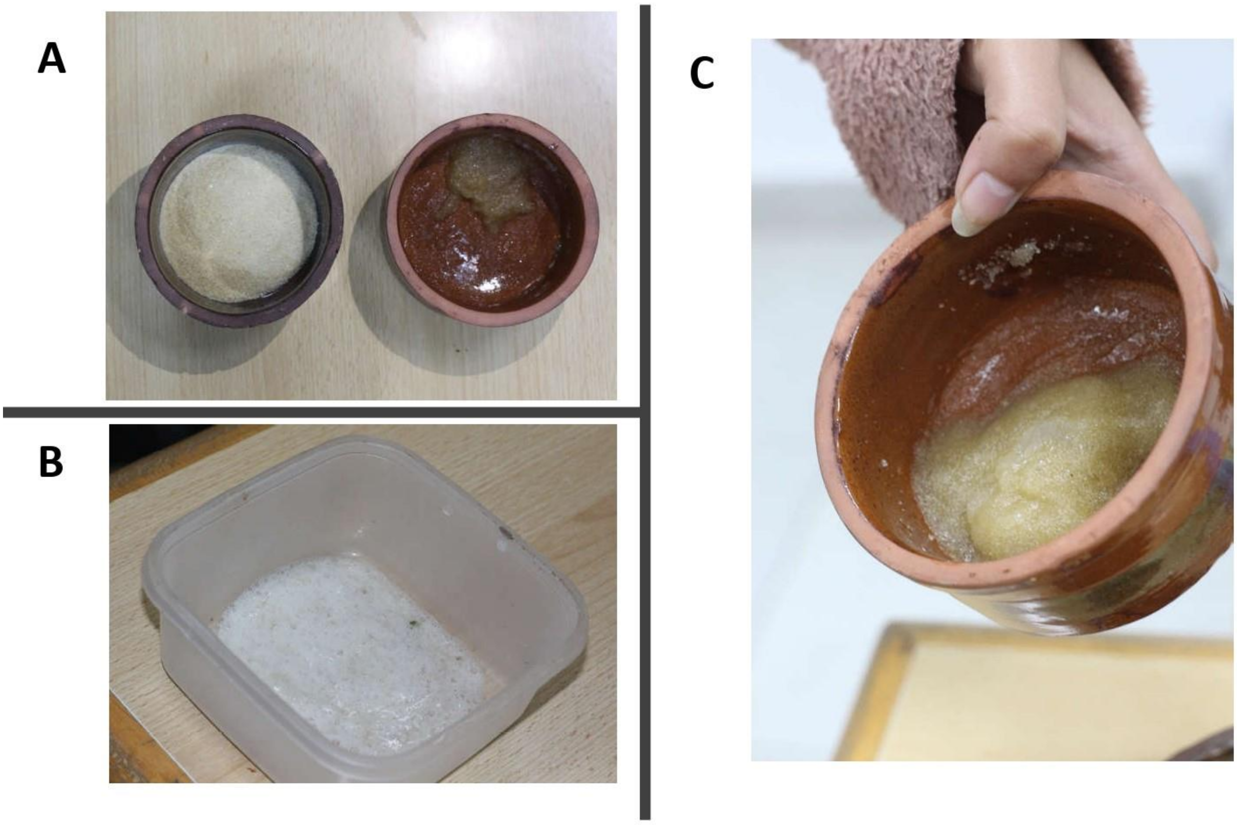

Fig. S7. Preparation of Aloe Vera – Gelatin mixture. (**A**) Gelatin powder before and after adding warm water. (**B**) Aloe Vera gel. (**C**) Aloe Vera Gel after adding Gelatin

S6 ImpEdded Skin Fabrication

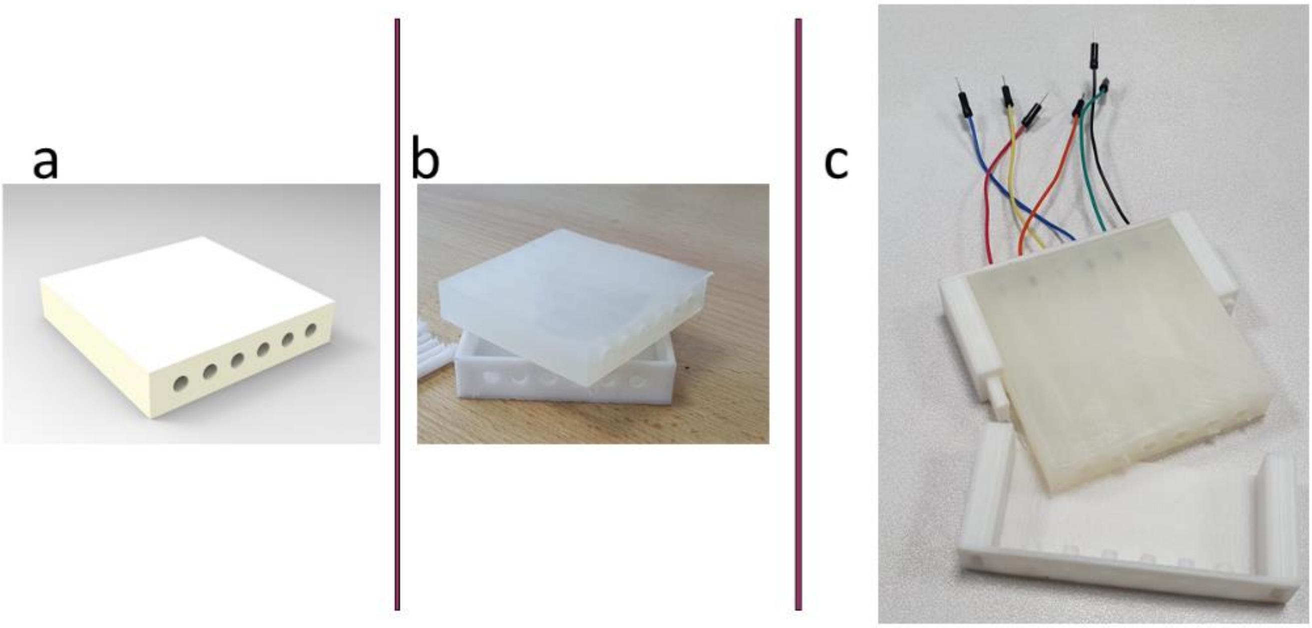

Fig. S8. Soft Skin Design and Fabrication. The ImpEdded skin simulation as in (**A**) and the mold casting is shown in (**B**). the assembly of the wires and the casing is shown in (**C**), the skin strap is filled with the mixture then tested.

The ImpEdded Skin was fabricated using one of the materials used in the first experiment; silicon rubber shore hardness 8. It was chosen owing to its better results. The skin strap was designed with six channels embedded in the soft structure of a round cylindrical shape and filled by a mixture of Aloe Vera and gelatin. The skin mold and skin holder is fabricated by the same way of the mold and the holder of the soft silicon structure used in the first experiment, both the mold and the holder are shown in fig. S8.
