# Supplementary material for: Tactile sensing biohybrid soft E-skin based on bioimpedance using aloe vera pulp tissues
Source: Sci Rep. 2021 Feb 4;11:3054. doi: 10.1038/s41598-021-82549-x (PMC7862233; doi:10.1038/s41598-021-82549-x)
Supplement: Supplementary file 2 — Supplementary Legend. [file 41598_2021_82549_MOESM2_ESM.pdf]

Manuscript title: ImpEdded Skin: Bioimpedance Augments Tactile Sensing for Bio-hybrid E-skin.

Authors: Mostafa A. Mousa, MennaAllah H. Soliman , Mahmood A. Saleh , and Ahmed G. Radwan

Video Caption: This video shows the experiment conducted to relate between applied force and bioimpedance expressed in terms of frequency. The source of force in this experiment is a double acting cylinder controlled by a pneumatic circuit. The cylinder is fixed to a mechanical setup where the silicone structure is placed. The value of applied force is acquired by the force sensor and change in bioimpedance is acquired in the form of change of output frequency of SRCO circuit. Both of them undergo two signal conditioning circuits before being acquired by the data acquisition circuit. The oscilloscope shows how the relation between the frequency and the amount of pressure exerted on the soft structure is proportional. The experiment is conducted by the author "Mostafa A. Mousa".
